# Exposure to Endocrine Disruptors and Nuclear Receptors Gene Expression in Infertile and Fertile Men from Italian Areas with Different Environmental Features

**DOI:** 10.3390/ijerph121012426

**Published:** 2015-10-05

**Authors:** Cinzia La Rocca, Sabrina Tait, Cristiana Guerranti, Luca Busani, Francesca Ciardo, Bruno Bergamasco, Guido Perra, Francesca Romana Mancini, Roberto Marci, Giulia Bordi, Donatella Caserta, Silvano Focardi, Massimo Moscarini, Alberto Mantovani

**Affiliations:** 1 Department of Food Safety and Veterinary Public Health, Istituto Superiore di Sanità, Roma 00161, Italy; E-Mails: sabrina.tait@iss.it (S.T.); luca.busani@iss.it (L.B.); brn_001@hotmail.it (B.B.); francesca.r.mancini@gmail.com (F.R.M.); alberto.mantovani@iss.it (A.M.); 2 Department of Physical, Earth and Environmental Sciences, University of Siena, Siena 53100, Italy; E-Mails: cristiana.guerranti@bsrc.it (C.G.); guido.perra.71@gmail.com (G.P.); focardi@unisi.it (S.F.); 3 Bioscience Research Center, Orbetello (GR) 58015, Italy; 4 Department of Obstetric and Gynecological Sciences and Urological Sciences, University of Roma Sapienza, S. Andrea Hospital, Roma 00189, Italy; E-Mails: ciardofrancesca@libero.it (F.C.); bordi.giulia@gmail.com (G.B.); donatella.caserta@uniroma1.it (D.C.); massimo.moscarini@uniroma1.it (M.M.); 5 Department of Biomedical Sciences and Advanced Therapies, Section of Obstetrics and Gynaecology, University of Ferrara, Ferrara 44124, Italy; E-Mail: roberto.marci@unife.it

**Keywords:** human exposure, men infertility, PFOS, PFOA, BPA, DEHP, MEHP, biomarkers

## Abstract

Internal levels of selected endocrine disruptors (EDs) (*i.e.*, perfluorooctane sulfonate (PFOS), perfluorooctanoic acid (PFOA), di-2-ethylhexyl-phthalate (DEHP), mono-(2-ethylhexyl)-phthalate (MEHP), and bisphenol A (BPA)) were analyzed in blood/serum of infertile and fertile men from metropolitan, urban and rural Italian areas. PFOS and PFOA levels were also evaluated in seminal plasma. In peripheral blood mononuclear cells (PBMCs) of same subjects, gene expression levels of a panel of nuclear receptors (NRs), namely estrogen receptor α (ERα) estrogen receptor β (ERβ), androgen receptor (AR), aryl hydrocarbon receptor (AhR), peroxisome proliferator-activated receptor γ (PPARγ) and pregnane X receptor (PXR) were also assessed. Infertile men from the metropolitan area had significantly higher levels of BPA and gene expression of all NRs, except PPARγ, compared to subjects from other areas. Subjects from urban areas had significantly higher levels of MEHP, whereas subjects from rural area had higher levels of PFOA in both blood and seminal plasma. Interestingly, ERα, ERβ, AR, PXR and AhR expression is directly correlated with BPA and inversely correlated with PFOA serum levels. Our study indicates the relevance of the living environment when investigating the exposure to specific EDs. Moreover, the NRs panel in PBMCs demonstrated to be a potential biomarker of effect to assess the EDs impact on reproductive health.

## 1. Introduction

Over recent decades, epidemiological studies have been reporting worrisome trends in the incidence of human infertility rates, implying a growing demand for assisted reproduction. The male infertility factors are estimated to account for 30% couples’ infertility, defined as “the failure to achieve a clinical pregnancy after 12 months or more of regular unprotected sexual intercourse” [1]. Indeed, the reference values for semen quality assessment have been recently updated by the World Health Organization (WHO) to improve diagnosis criteria [2]. Noticeably, for many providers, semen analysis is the only marker responsible for many of the couple referrals to fertility clinics, without paying attention to etiology or risk factors [3]. In the meanwhile, a growing number of studies associate the human exposure to specific chemicals identified as endocrine disruptors (EDs), as detected by their presence in body fluids, with a vast array of reproductive disorders in both sexes [4]. In particular, the widespread decline in semen quality may be a long-term consequence of altered reproductive programming (so called Testicular Dysgenesis Syndrome), as well as consequence of continuous exposure to EDs in the living environment [5,6,7].

Extensive detection of industrial chemicals in human serum, seminal plasma and follicular fluid has led the scientific community to hypothesize that these compounds may disrupt hormonal homoeostasis leading to a variety of physiological impairments, mainly on reproductive health, but also on thyroid function and related neurodevelopmental effects, homeostasis of fat and glucose metabolism as well as increased susceptibility to some cancers [7].

Di-2-ethylhexyl phthalate (DEHP) and Bisphenol A (BPA) are non-persistent EDs mainly used as plasticizers, which are widely present in foods, living environment and consumer products. DEHP is used primarily in soft polyvinyl chloride products (PVC) such as building materials, electronic devices floorings, clothing, furniture, food contact materials, and personal care products. Upon intake, DEHP is quickly metabolized to its major toxic metabolite, mono-(2-ethylhexyl) phthalate (MEHP), representing the toxicologically relevant biomarker of DEHP exposure [8]. BPA is used in food containers (bottles, microwave ovenware, and linings for canned foods and beverages) but also in non-food items, including epoxy-resin based paints, PVC medical devices, surface coatings, thermal paper, parts of electronic products, and flame retardants [9]. The perfluorinated EDs, such as perfluorooctane sulfonate (PFOS) and perfluorooctanoic acid (PFOA), are more persistent than DEHP and BPA; although their use in consumer products is currently restricted, they have been used in a wide range of industrial products such as textiles, paper, cleaning agents paints and insecticide formulations, leading to their widespread presence in the environment. They also concentrate in food chains, especially in fish [10].

Several studies have reported the association of exposure to phthalates and BPA with impaired semen quality as well as steroid and thyroid hormone levels in infertile men [11,12,13]. PFOS and PFOA exposure have also been associated with poor semen quality [14,15]. Experimental studies show that all these EDs interact with several nuclear receptors (NRs): PFOS/PFOA enhance the activity of the estrogen receptors (ERs) [16], the peroxisome proliferator-activated receptors (PPARs) [17,18] and the pregnane X receptor (PXR) [19]; DEHP and MEHP are agonists of PPARs and PXR [20,21,22,23].

BPA is considered mainly as an ERα and ERβ agonist but it can also affect other endocrine pathways, e.g., by acting as antagonist of the androgen receptor (AR) or as agonist of the aryl hydrocarbon receptor (AhR), involved in cross talk processes with ERs, AR and other NRs, and of PXR [24,25,26].

In the frame of the PREVIENI project (“Study in model areas on the environmental and health impact of some emerging chemical contaminants (endocrine disrupters): living environment, reproductive outcomes and repercussions in childhood”), we investigated in fertile and infertile subjects of both sexes the internal exposure levels of PFOS, PFOA, BPA, DEHP and its metabolite MEHP, as well as the gene expression of ERα, ERβ, AR, PPARγ, AhR and PXR, as potential biomarkers of effect. Both biomarkers of exposure and effect were studied in blood, since contaminants can directly affect NRs expression in peripheral blood mononuclear cells (PBMCs), relevant for their physiological significance. Indeed, these cells may represent one of the first targets possibly affected by EDs exposure as reported in both *in vitro* [27] and human population studies [28,29]. Blood is considered the matrix of choice for compounds like PFOS and PFOA, but not for BPA and phthalates, which are regarded as EDs undergoing quick metabolism [30]. Nevertheless, several recent studies in humans measured BPA and/or MEHP in serum evidencing their presence into the bloodstream addressing possible environment-health associations, such as the potential relationships with reproductive function and hormonal balance [31], diabetes [32], coronary risk [33] and hypertension [34].

Subjects were enrolled in three Italian areas representing different living environment scenarios, which may be related to different EDs exposure patterns: Rome (Lazio, Central Italy), with all of the features of a metropolitan environment and lifestyle; Ferrara (Emilia-Romagna, Northern Italy), a medium-sized town amid a prosperous area with many farms and small- or medium-sized industries; Sora (Lazio, Central Italy), a rural municipality characterized by intensive agricultural activities. Previously, we published PREVIENI data on women showing that the area of residence can be relevant for exposure assessment of EDs and that modulation of selected NRs expression (ERα, ERβ, AR, AhR, and PXR) may represent a useful biomarker for reproductive disorders and EDs exposure [29].

This work presents data on fertile and infertile men from the tree areas under study showing EDs internal levels measured in whole blood (PFOS and PFOA) or in serum (BPA and DEHP) and in seminal plasma (PFOS and PFOA) as well as NRs gene expression in PBMCs. The aim is to: assess whether the area of residence can be related to a different exposure scenario; evaluate the possible association between male infertility and biomarkers of exposure to specific EDs; and estimate the correlation between EDs exposure and NRs gene expression, to define, as final goal, a panel of biomarkers of effect related to biomarkers of exposure.

## 2. Methods

### 2.1. Areas Characterization

Three different geographic areas were considered in this study: metropolitan area (Rome, Lazio Region, Central Italy); medium-sized urban area (Ferrara, Emilia-Romagna Region, Northern Italy); rural area (Sora, Lazio Region, Central Italy). The three areas were chosen by considering territorial, demographic, and productive (number and percentage of industries by category of production per km^2^) indicators due to their potential contribution to the environmental contamination as regards the EDs considered. Data on the selected indicators for each area were obtained for the year 2011 from the Italian National Institute of Statistics (ISTAT) and are reported in Table 1 and in our previous study [29].

**Table 1:** Distribution of a set of territorial, demographic and productive indicators in the study areas. Data from the Italian National Institute of Statistics (ISTAT).

Areas	Metropolitan (Rome)		Urban (Ferrara)		Rural (Sora)	
Indicators	1–10 employees	>10 employees	1–10 employees	>10 employees	1–10 employees	>10 employees
Agricultural enterprises	393	17	1684	10	4	0
Textile industries	206	9	40	2	4	0
Petroleum refinery	16	12	0	1	0	0
Manufactures of chemicals	121	43	12	8	4	0
Manufactures of articles of rubber	0	0	0	0	0	0
Manufacture of articles of plastics	0	0	0	0	0	0
Sanitation and waste management	39	6	2	0	0	0
Population	2,724,347		134,464		26,542	
Surface (km^2^)	1307.71		404.36		71.82	
Population density (inhabitants/km^2^)	2083.30		332.54		369.56	

### 2.2. Study Subjects

From January 2009 to December 2011, on a voluntary basis, 70 infertile men were enrolled in the study in the following medical centers per area:

• *n* = 28: Department of Women Health and Territorial Medicine of “Sapienza” University “Sant’Andrea” Hospital, Rome;
• *n* = 19: Department of Biomedical Sciences and Advanced Therapies, Section of Obstetrics and Gynaecology, University of Ferrara; and
• *n* = 23: Infertility Center Sterility Therapy and Study of Sora.

Inclusion criteria were: residing in the municipalities included in the area, age from 27 to 40 years, body mass index (BMI) < 30 and PBMCs levels within the range of normal values for age and sex.

Infertile men affected by non-genetic oligospermia (decreased number of spermatozoa in semen) and/or reduced sperm motility, or by idiopathic infertility were enrolled in the study. Occupational exposure to the selected EDs (plastic, housewares or textile industries), smoking habit, vegetarian diet, azoospermia, associated varicocele, any genital surgery including corrective surgery for undescended testis, a history of previous epididymo-orchitis or prostatitis, history of cancer chemotherapy, testosterone and antiandrogens treatment, and eukocytospermia, were considered as exclusion criteria. Aged- and BMI-matched fertile men from Rome (*n* = 34), Ferrara (*n* = 41) and Sora (*n* = 8) were enrolled as controls in the same centers. They were men with normal reproductive function who obtained a spontaneous pregnancy and had healthy babies in the last year. The study has been carried out in accordance with The Code of Ethics of the World Medical Association (Declaration of Helsinki). Approval from the ethical committees of the responsible structures of the collaborating medical centers were obtained before the beginning of this study and all patients gave informed consent to study inclusion.

### 2.3. Collection and Storage of Samples

Blood and seminal plasma samples were collected for each subject enrolled in the study, with the exception of ten subjects enrolled as controls in the area of Rome that provided exclusively blood samples. Glass vials were used in order to avoid possible release of DEHP or BPA from plastics. Three aliquots of venous blood were collected from each man. For EDs level determination, 5 mL of heparin-treated whole blood and 10 mL centrifuged blood to obtain serum were sampled and sent to the Environment Science Department “G. Sarfatti” (now Department of Physical, Earth and Environmental Sciences) of the University of Siena.

For NRs gene expression evaluation in PBMCs, two different blood sampling methods were adopted, depending on the sampling site, in order to avoid RNA denaturation: blood samples from Rome were collected in heparin tubes and processed within 48 h, whereas samples from Ferrara and Sora were collected in PAXgene Blood RNA Tubes (PreAnalitiX, Plymouth, UK) and frozen until use. All samples were sent to the Food and Veterinary Toxicology Unit (Istituto Superiore di Sanità, Rome).

All semen samples were collected in wide-mouthed sterile container by masturbation after 3–5 days of sexual abstinence. All samples were kept at 37 °C and examined immediately after complete liquefaction. Only one sample per patient was included in the study.

### 2.4. Chemical Analysis of Biomarker of Exposure

Based on established literature methods, BPA, DEHP and MEHP were measured in serum, PFOS and PFOA in whole blood and in seminal plasma.

#### 2.4.1. PFOS/PFOA

Details for the analysis of PFOS/PFOA in whole blood were previously reported [29,35]. Chemical analysis of seminal plasma was performed following the same procedure adopted for whole blood and yet tested for these and other body fluids [15]. Briefly, the samples were homogenized and extracted with methyl tert-butyl ether (MTBE, J.T. Baker, Center Valley, PA, USA). The solvent was evaporated under nitrogen and replaced with methanol (J.T. Baker). Quantification was performed in a HPLC (equipped with Betasil^©^ C18 column, Thermo Electron Corporation, San Jose, CA, USA) interfaced to a mass spectrometer at linear triple quadrupoles, by electrospray ionization (ESI) source, working in negative ion mode (Finnigan LTQ Thermo Electron Corporation, San Jose, CA, USA). The limit of detection (LOD) for both PFOS and PFOA were 0.4 ng/mL, corresponding to the value of the compounds in the blanks +3 Standard Deviation (SD).

#### 2.4.2. DEHP/MEHP

The analytical procedure for the extraction of DEHP and MEHP from serum samples has been previously described [29,35]. Briefly, 0.5 g of each thawed sample were added with 4 mL of acetone (J.T. Baker), sonicated and centrifuged for 15 min at 3000 rpm for two times. Supernatants were evaporated in a centrifugal evaporator (Thermo Scientific, Waltham, Massachusetts, MA, USA) and suspended with 0.5 mL of deionized water and 4 mL of acetic acid (J.T. Baker). Quantification was performed by LC-ESI-MS system, equipped with a reverse phase HPLC column (Wakosil3C18, 2.0 × 100 mm, 3 μm; Wako Pure Chemical Industries Ltd., Richmond, VA, USA), operating in negative (MEHP) or positive (DEHP) ion mode. The LODs were 2 ng/mL for MEHP and 10 ng/mL for DEHP.

#### 2.4.3. BPA

Total BPA in serum was analyzed according to the procedure previously described [29,35]. Before extraction with ethyl ether (J.T. Baker), each aliquot of 0.5 mL of serum was incubated with 2 μL/mL of the enzyme I glucuronidase (Sigma-Aldrich, Saint Louis, MO, USA) at 37 °C for 12 h. After centrifugation, the collected supernatants were evaporated and reconstituted in 0.5 mL of methanol. HPLC-ESI-MS instrument, equipped with a Betasil C18 column 50 × 2.1 mm operated in negative ion mode. The identification of BPA was obtained by fragmentation of the ion 227 with collision energy of 35 and production of the ion (m/z) 212. The ESI source was set at a voltage of 5 kV and to a rush of 3 μA. The LOD was 0.5 ng/mL.

#### 2.4.4. Data Quality Assurance and Quality Control

Measures to avoid contamination from plasticizers in test materials included the use of metal needles and glassware vials for collection and storage of samples, the use of glass labware rinsed by acetone and hexane to remove potential contaminants and the assessment of method blanks. Data quality assurance and quality control protocols for all compounds included matrix spikes, laboratory blanks, and continuing calibration verification. Blanks were analyzed with each set of five samples as a check for possible laboratory contamination and interferences: levels of chemicals in such samples resulted below the limit of detection for each compound.

### 2.5. Gene Expression Analysis of Nuclear Receptors

Procedures for RNA extraction and gene expression analysis of NRs were previously reported [29]. Briefly, heparin-treated blood samples obtained by subjects from Rome were processed using Lympholyte^®^-H density gradient separation medium (Cederlane Laboratories Limited, Ontario, Canada) to isolate PBMCs according to manufacturer’s instructions. Total RNA was then extracted by the RNeasy Mini Kit (Qiagen, Hilden, Germany). Blood samples from Ferrara and Sora, collected in PAXgene RNA content was extracted by the PAXgene Blood RNA Kit (Qiagen, Hilden, Germany). For each sample, total RNA was quantified by NanoDrop (Thermo Scientific Wilmington, DE, USA) and assessed for its integrity by 1% agarose gel electrophoresis. From each sample, 1 μg of RNA was reverse transcribed to cDNA by the cDNA Synthesis Kit (Quantace, London, UK). Gene expression analysis was performed by quantitative Real time polymerase chain reaction (PCR) using the Sensi Mix SYBR Kit (Quantace, London, UK). As reference gene, we assessed the stability of beta-actin and glyceraldehyde-3-phosphate dehydrogenase (GAPDH) on a limited sub-group of fertile and infertile individuals among the first sampled. Since the stability of beta-actin was much lower than that of GAPDH, we chose to use only the latter as reference gene. Indeed, mean cycle threshold (Ct) value for all the men analyzed in the three areas was 20.3 with a standard deviation of 1.2, which we considered acceptable given the number of subjects and the inter-individual variability. The list of the specific primers for the selected NRs and GAPDH were published in the previous report [29]. Real-time PCR reactions were run on a Stratagene MP3005P Thermocycler (Stratagene, La Jolla, Ca, USA).

Data from Real-time PCR experiments are expressed as 2^−ΔCt^ values (ΔCt = Ct_TG_ – Ct_RG_, where TG = target gene; RG = reference gene) for both control and infertile individuals instead of 2^−ΔΔCt^, as generally performed, to avoid loss of information of controls which, otherwise, would have been flattened to 1.

Seven samples from infertile subjects (three from Rome, three from Ferrara and one from Sora) plus eight control samples (four from Rome and four from Ferrara) were not analyzed for NRs gene expression due to delivery problems.

### 2.6. Statistical Analysis

We performed statistical descriptive and comparative analysis using non-parametric tests. We decided to limit the inference to single variable analysis (univariate statistics) stratifying by areas to provide unbiased results, taking into account the number of samples.

The concentration of each ED below the respective LOD has been considered as “<LOD” for the statistical analyses. For the inferential analyses and comparisons the value below LOD has been replaced by half the LOD value (medium-bound). Dichotomous variables for concentrations of the EDs (0 if ≤ LOD and 1 if > LOD) were created. EDs concentrations in blood, PFOA and PFOS concentrations in semen and NRs expression values were not normally distributed and the log transformation did not normalize the distributions. Therefore, the differences of EDs internal exposures related to the health status (infertile *vs.* fertile men) and the area of residence (rural, urban or metropolitan) were assessed with the Wilcoxon Mann-Whitney test, adjusting for multiple comparisons using Bonferroni procedure for correcting the *p*-value, which for each individual test was set at 1/5 of the level of statistical significance chosen.

The risk of infertility in relation to the EDs concentration in blood and to PFOA and PFOS concentration in semen was calculated comparing infertile and fertile subjects by the exposure (as explained above) using univariate analysis and stratifying by area of residence. Chi-square was calculated for each test according to Mantel-Haenszel.

Correlation between EDs concentrations in blood with the PFOA and PFOS concentrations in semen was assessed with a Spearman’s rank correlation test with Bonferroni correction, separately among cases and controls. The same test was run to investigate the correlation between EDs concentrations in blood with the NRs expression.

Statistical analysis was performed with STATA 11.2 (StataCorp, 4905 Lakeway Drive, College 17 Station, Texas, TX, USA) setting significance at *p* < 0.05 for all the statistical tests performed.

## 3. Results

### 3.1. Study Population

The mean age and BMI of enrolled men in the three areas are summarized in Table 2. No significant difference was observed between infertile and fertile subjects (data not shown), according to age- and BMI-matching criteria used for enrollment.

**Table 2:** Age (mean and range) and body mass index (BMI) (mean ± SD) of men enrolled in the three areas.

Areas	Age	BMI
Metropolitan (*n* = 62)	37.2 (32–40)	25.0 ± 3.1
Urban (*n* = 60)	34.02 (30–40)	25.2 ± 3.0
Rural (*n* = 31)	35.6 (27–40)	26.3 ± 2.9

*n*, number of subjects enrolled in each area; BMI, body mass index.

### 3.2. Biomarkers of Exposure

PFOS, PFOA, MEHP and BPA serum/blood levels are summarized in Table 3 (serum/blood). The results, expressed as mean, median and interquartile range (25th–75th percentile) values, are provided for both fertile and infertile groups by area.

The percentage of subjects exposed to each specific ED, by considering the numbers of subjects in which the levels were above the limit of detection (LOD), varied considerably among the three areas under study. Since DEHP was found above the LOD only in four infertile men in the rural area (three subjects levels in the range 14.04–21.33 ng/mL; one subject level: 112.40 ng/mL) it was excluded from the analysis. PFOA was detected in over 75% of the subjects in the rural area compared to the urban (47%) and metropolitan (7%) areas, whereas PFOS was detected in about 30% of the subjects in each area. MEHP was detected in about 60% of subjects from the urban area, compared to 20% and less than 10% in the metropolitan and rural areas, respectively. BPA was detected in over 60% of subjects from the metropolitan area, compared to about 30% and only 3%, in the urban and rural areas, respectively (Table 3).

Next, we compared the blood/serum median concentration levels of EDs among infertile and fertile men residing in different areas. Similar exposure patterns were observed in the two groups: PFOA levels increased significantly from the metropolitan (the lowest), to the urban, up to the rural area (the highest, with 5.59 ng/mL blood and 4.9 ng/mL blood in infertile and fertile subjects, respectively). BPA levels were significantly higher in the metropolitan area (infertile: 19.7 ng/mL serum; fertile: 4.1 ng/mL serum) than in the urban and, especially, the rural areas. MEHP levels were significantly higher in men from the urban area (infertile: 2.8 ng/mL serum; fertile: 4.2 ng/mL serum) with respect to men in metropolitan and rural areas, which presented comparable levels.

The comparison of EDs internal levels between infertile and fertile men highlighted that, when considered together irrespective of area of residence, fertile men had significantly higher levels of MEHP. Such difference was no more present when data were stratified by area. In the meanwhile, stratification by area put into evidence significant differences between infertile and fertile subjects in the metropolitan area, where infertile men had significantly higher BPA and PFOS levels compared to fertile men (Table 3).

**Table 3:** Analytical values of PFOS, PFOA (ng/mL blood), MEHP and total BPA (ng/mL serum) in enrolled men grouped by area of residence and subject group.

Chemicals		PFOA		PFOS		MEHP		BPA	
**Areas**		*infertile*	*fertile*	*infertile*	*fertile*	*infertile*	*fertile*	*infertile*	*fertile*
Total	mean	2.3	1.8	3.8	1.1	2.9	5.6	9.3	5.7
(70 infertile;	median	<0.4	<0.4	<0.4	<0.4	<2.0 *	<2.0 *	<0.5	<0.5
83 fertile)	25th *p* ^#^	<0.4	<0.4	<0.4	<0.4	<0.2	<2.0	<0.5	<0.5
	75th *p*	4.4	2.9	1.6	0.8	<2.0	6.7	16.8	7.9
	%>LOD	47.10%	45.80%	32.90%	26.50%	24.30%	42.20%	42.90%	33.70%
Metropolitan	mean	0.5	0.5	8.1	1.6	3.6	5.5	19.2	11.2
(28 infertile;	median	<0.4 ^a^	<0.4 ^a^	<0.4 *	<0.4 *	<2.0 ^a^	<2.0 ^a^	19.7 ^a,^*	4.1 ^a,^*
34 fertile)	25th *p*	<0.4	<0.4	<0.4	<0.4	<2.0	<2.0	3.2	<0.5
	75th *p*	<0.4	<0.4	15.9	<0.4	3.3	<2.0	33.1	21.7
	%>LOD	7.10%	8.80%	42.90%	23.50%	17.90%	20.60%	75.00%	50.00%
Urban	mean	1.8	2.5	0.5	1	3.8	6.4	2.8	2.2
(19 infertile;	median	<0.4 ^b^	2.4 ^b^	<0.4	<0.4	2.8 ^b^	4.2 ^b^	<0.5 ^b^	<0.5 ^b^
41 fertile)	25th *p*	<0.4	<0.4	<0.4	<0.4	<2.0	<2.0	<0.5	<0.5
	75th *p*	3.6	4.0	0.5	0.7	5.9	8.1	5.5	4.6
	%>LOD	47.40%	70.70%	26.30%	26.80%	57.90%	65.90%	42.10%	26.80%
Rural	mean	5.2	4.3	1.2	1	<2.0	1.5	2.6	<0.5
(23 infertile;	median	5.5 ^c^	4.9 ^b^	<0.4	<0.4	<2.0 ^a^	<2.0 ^a^	<0.5 ^c^	<0.5 ^b^
8 fertile)	25th *p*	4.4	2.1	<0.4	<0.4	<2.0	<2.0	<0.5	<0.5
	75th *p*	6.1	6.6	0.6	1.3	<2.0	<0.2	<0.5	<0.5
	%>LOD	95.70%	75.00%	26.10%	37.50%	4.40%	12.50%	4.40%	0.00%

LOD = 0.4 ng/mL for PFOS and PFOA; 2 ng/mL for MEHP; 0.5 ng/mL for BPA. * indicates statistically significant different values between fertile and infertile men in the same area of residence (Mann-Whitney test corrected with the Bonferroni procedure). ^a,b,c^ Different superscript letters indicate statistically significant different values between areas within subjects of the same group (Mann-Whitney test corrected with the Bonferroni procedure). ^#^ 25th and 75th *p* indicate percentile values. PFOA, perfluorooctanoic acid; PFOS, perfluorooctane sulfonate; MEHP, mono-(2-ethylhexyl)-phthalate; BPA, bisphenol A.

The concentrations of perfluorinated EDs in seminal plasma are summarized in Table 4. The results, expressed as mean, median and interquartile range (25th–75th percentile) values, are provided for both fertile and infertile groups by area. PFOA was detected in more than 75% of subjects from the rural area and in about 30% from the urban area while it was not detected in subjects from the metropolitan area. Otherwise, PFOS was detected in 20% of the subjects from the urban and rural areas and in less than 10% of the subjects from the metropolitan area (Table 4).

Similarly to what was observed in blood, PFOA seminal plasma levels of infertile men residing in the rural area showed the highest levels (Table 4). Such difference was also observed, to a lower extent, in fertile men where PFOA seminal plasma levels were higher in the rural area compared to metropolitan and urban areas, which had comparable levels. No significant difference was highlighted between infertile and fertile subjects, in any area. PFOS concentration in the semen did not show any difference neither by area nor between infertile and fertile subjects (Table 4).

**Table 4:** Analytical values of PFOS and PFOA (ng/mL semen) in enrolled men grouped by area of residence and subject group.

Chemicals		PFOA		PFOS	
**Areas**		***infertile***	***fertile***	***infertile***	***fertile***
Total	mean	1.6	1.2	1.1	1.1
(70 infertile;	median	<0.4	<0.4	<0.4	<0.4
73 fertile)	25th *p* ^#^	<0.4	<0.4	<0.4	<0.4
	75th *p*	3.1	2.4	<0.4	<0.4
	%>LOD	32.90%	32.90%	18.60%	11.00%
Metropolitan	mean	<0.4	<0.4	1.2	1.7
(28 infertile;	median	<0.4 ^a^	<0.4 ^a^	<0.4	<0.4
24 fertile)	25th *p*	<0.4	<0.4	<0.4	<0.4
	75th *p*	<0.4	<0.4	<0.4	<0.4
	%>LOD	0.00%	0.00%	7.10%	8.30%
Urban	mean	1	1.5	1.4	1
(19 infertile;	median	<0.4 ^b^	<0.4 ^b^	<0.4	<0.4
41 fertile)	25th *p*	<0.4	<0.4	<0.4	<0.4
	75th *p*	<0.4	2.7	3.1	<0.4
	%>LOD	21.10%	43.90%	26.30%	14.60%
Rural	mean	3.7	2.5	0.8	<0.4
(23 infertile;	median	3.4 ^c^	3.2 ^b^	<0.4	<0.4
8 fertile)	25th *p*	2.4	1.3	<0.4	<0.4
	75th *p*	5.5	3.5	1.5	<0.4
	%>LOD	82.60%	75.00%	26.10%	0.00%

LOD = 0.4 ng/mL for PFOS and PFOA; ^a,b,c^ Different superscript letters indicate statistically significant different values between areas within subjects of the same group (Mann-Whitney test corrected with the Bonferroni procedure). ^#^ 25th and 75th *p* indicate percentile values. PFOA, perfluorooctanoic acid; PFOS, perfluorooctane sulfonate.

Consistent with the above finding, PFOA semen and blood concentration levels were highly correlated in the infertile group (Rho = 0.59; *p* < 0.001), while no correlation was found in the fertile group (Rho = 0.08; *p* = 1) (data not shown). No correlation was detected in either infertile or fertile groups between PFOS semen and blood levels.

Finally, we assessed whether the EDs under investigation could be identified as risk factors for male infertility based on measured internal exposure levels. Grouping together the subjects from the three areas, a significant negative association was found between MEHP serum levels and infertility (odds ratio = 0.4, with 95% confidence limits 0.2-0.9). However, when stratifying by area of residence, no significant association was found either for MEHP or for the others EDs (data not shown).

### 3.3. Nuclear Receptors Gene Expression

NRs gene expression levels (mean, median and interquartile range) in infertile and fertile men by area are summarized in Table 5.

The mRNAs of the selected NRs were detected in all samples examined, therefore confirming the plausibility of the panel. Expression levels in PBMCs were approximately comparable for ERα, ERβ, AR and PXR, while AhR and PPARγ were expressed at lower levels.

Expression levels of ERα, ERβ, AR, AhR and PXR were significantly higher in infertile subjects from the metropolitan area compared to the other two areas, showing the pattern metropolitan > urban > rural areas. The median expression levels in men from the metropolitan area were about four-(PXR) to eight-fold (ERα) higher than in men from the urban area and about 15-(AhR) to 492-fold (PXR) higher than those from the rural area. Similar significant differences were observed in fertile men as regards ERα and PXR with the same pattern metropolitan > urban > rural areas. More evident differences in median expression levels were observed between men from metropolitan and urban areas (about 28- and 46-fold for ERα and PXR, respectively) and still large difference between metropolitan and rural residing men (about 55- and 162-fold for ERα and PXR, respectively). ERβ, AR and AhR expression was significantly higher in fertile men from the metropolitan area compared to men from both urban and rural areas, which were comparable, with difference ranging from about three-(AhR) to 39-fold (ERα) in expression levels. No differences were detected for PPARγ in either areas or infertile and fertile men.

A difference between infertile and fertile subjects was observed only for AhR, its expression being significantly higher in infertile men from the metropolitan area compared to fertile subjects of the same area (Table 5).

The analysis of the correlation between EDs concentration and the NRs expression in blood evidenced that, in both infertile and fertile men, PFOA levels were negatively correlated to the expression of ERα, ERβ, AR, AhR and PXR. On the contrary, BPA serum levels were positively correlated with the same NRs. No correlation was found with PPARγ (Table 6).

**Table 5:** Gene expression values of nuclear receptors in enrolled men grouped by area of residence and subject group. Data are expressed as 2^−ΔCt^ values with GAPDH as reference gene.

Nuclear Receptors		ERα		ERβ		AR		PPARγ		AhR		PXR	
**Areas**		*infertile*	*fertile*	*infertile*	*fertile*	*infertile*	*fertile*	*infertile*	*fertile*	*infertile*	*fertile*	*infertile*	*fertile*
Total	mean	0.0658	0.0717	0.0551	0.0667	0.0314	0.0354	0.0003	0.0007	0.0049	0.0036	0.0635	0.0684
(63 infertile;	median	0.0091	0.0013	0.0079	0.0031	0.0045	0.0016	0.0002	0.0002	0.0016	0.0013	0.0071	0.0008
75 fertile)	25th *p* ^#^	0.0005	0.0004	0.0011	0.0008	0.0007	0.0004	0.0001	0.0001	0.0006	0.0007	0.0002	0.0002
	75th *p*	0.0561	0.0511	0.0457	0.0544	0.0274	0.0260	0.0003	0.0003	0.0080	0.0040	0.0509	0.0525
Metropolitan	mean	0.1511	0.1595	0.1278	0.1487	0.0718	0.0774	0.0002	0.0002	0.0108	0.0046	0.1338	0.1492
(25 infertile;	median	0.0629 ^a^	0.0277 ^a^	0.0550 ^a^	0.0391 ^a^	0.0324 ^a^	0.0157 ^a^	0.0001	0.0002	0.0090 ^a,^*	0.0035 ^a,^*	0.0492 ^a^	0.0325 ^a^
30 fertile)	25th *p*	0.0209	0.0006	0.0172	0.0013	0.0092	0.0004	0.0000	0.0001	0.0035	0.0009	0.0157	0.0004
	75th *p*	0.2044	0.1170	0.1684	0.0788	0.0967	0.0527	0.0003	0.0003	0.0121	0.0052	0.2229	0.2094
Urban	mean	0.0223	0.0159	0.0156	0.0144 ^b^	0.0103	0.0089	0.0007	0.0049	0.0018	0.0034	0.0408	0.0162
(16 infertile;	median	0.0081 ^b^	0.0010 ^b^	0.0084 ^b^	0.0019	0.0055 ^b^	0.0013 ^a,b^	0.0001	0.0002	0.0013 ^b^	0.0011 ^b^	0.0136 ^b^	0.0007 ^b^
37 fertile)	25th *p*	0.0009	0.0005	0.0018	0.0007	0.0008	0.0005	0.0001	0.0001	0.0006	0.0005	0.0004	0.0003
	75th *p*	0.0343	0.0093	0.0274	0.0066	0.0174	0.0042	0.0011	0.0004	0.0026	0.0033	0.0564	0.0134
Rural	mean	0.0005	0.0005	0.0013	0.0013	0.0008	0.0007	0.0003	0.0002	0.0006	0.0010	0.0003	0.0002
(22 infertile;	median	0.0003 ^c^	0.0005 ^c^	0.0010 ^c^	0.0010 ^b^	0.0005 ^c^	0.0005 ^b^	0.0002	0.0002	0.0006 ^c^	0.0008 ^b^	0.0001 ^c^	0.0002 ^c^
8 fertile)	25th *p*	0.0002	0.0002	0.0006	0.0005	0.0003	0.0002	0.0001	0.0001	0.0003	0.0005	0.0001	0.0001
	75th *p*	0.0006	0.0006	0.0019	0.0013	0.0011	0.0011	0.0003	0.0003	0.0009	0.0015	0.0004	0.0002

* Indicates statistically significant different values between infertile and fertile men in the same area of residence (Mann-Whitney test corrected with the Bonferroni procedure). ^a,b,c^ Different superscript letters indicate statistically significant different values between areas within men of the same group (Mann-Whitney test corrected with the Bonferroni procedure). ^#^ 25th and 75th *p* indicate percentile values. ERα, estrogen receptor α; ERβ, estrogen receptor β; AR, androgen receptor; PPAR γ, peroxisome proliferator-activated receptor γ; AhR, aryl hydrocarbon receptor; PXR, pregnane X receptor.

**Table 6:** Correlation between the endocrine disruptors concentration in blood/serum and nuclear receptors gene expression in infertile and fertile subjects (Spearman’s rank correlation test, Bonferroni corrected). (**a**) Fertile subjects; (**b**) Infertile subjects.

(**a**)				
**Chemicals**	**PFOA**	**PFOS**	**MEHP**	**BPA**
**Nuclear Receptors**				
ERα	–0.51 *	0.04	–0.17	0.53 *
ERβ	–0.46 *	0.02	–0.2	0.51 *
AR	–0.49 *	0.02	–0.18	0.53 *
AhR	–0.31 *	0.19	–0.23	0.42 *
PXR	–0.52 *	0.08	–0.14	0.61 *
PPARγ	0.12	0.22	0.04	–0.34
(**b**)				
**Chemicals**	**PFOA**	**PFOS**	**MEHP**	**BPA**
**Nuclear Receptors**				
ERα	–0.76 *	0.04	0.02	0.51 *
ERβ	–0.75 *	0.04	0.03	0.49 *
AR	–0.74 *	0.03	0.01	0.50 *
AhR	–0.69 *	0.20	–0.02	0.50 *
PXR	–0.76 *	0.05	0.07	0.50 *
PPARγ	0.34	–0.19	–0.09	0.33

* Indicates statistically significant correlation. PFOA, perfluorooctanoic acid; PFOS, perfluorooctane sulfonate; MEHP, mono-(2-ethylhexyl)-phthalate; BPA, bisphenol A; ERα, estrogen receptor α; ERβ, estrogen receptor β; AR, androgen receptor; PPAR γ, peroxisome proliferator-activated receptor γ; AhR, aryl hydrocarbon receptor; PXR, pregnane X receptor.

## 4. Discussion

This study investigated EDs internal levels and gene expression levels of a panel of NRs in infertile and fertile men residing in metropolitan, urban and rural areas; the potential association between male infertility and internal levels of specific EDs and the association between EDs and NRs expression levels were also evaluated.

The three areas (Rome, Ferrara and Sora) included in this study represent quite distinct living environment scenarios according to selected territorial, demographic and productive indicators. Briefly, the metropolitan area was characterized by high population density and also included a number of industrial and even agricultural enterprises, the urban area showed the highest proportion of enterprises with more than 10 employees, while in the rural area neither factories nor farms with more than 10 employees were reported. Indeed, analyses of biomarkers of exposure and effects in the population of men under study highlighted relevant differences according to the area of residence.

The subjects from the metropolitan area are characterized by higher internal levels of BPA as well as gene expression of ERα, ERβ, AR AhR and PXR; moreover, only in this area infertile men had higher BPA and PFOS levels, as well as AhR expression, compared to fertile subjects.

The significantly higher levels of BPA in men from metropolitan area may reflect the greater presence of economic activities employing these chemicals, as well as characteristic usage patterns of food commodities and consumer products, suggesting a repeated and continuous uptake of the compound from aggregate exposure. Although, in the same area, statistically higher concentrations of BPA and PFOS were detected in infertile compared to fertile subjects, the exposure to BPA and PFOS did not represent risk factors for infertility. Previous studies reported an inverse association between BPA internal levels in men and semen quality parameters, such as sperm concentration, sperm motility, sperm vitality, used to assess infertility: in male partners of sub-fertile couples [12]; in workers exposed to BPA [36] and in young men from the general population [37]. For PFOS exposure, a negative association with semen quality was also reported in the general population [38].

The overexpression of AhR we observed in infertile subjects has been previously reported in Sertoli, Leydig and spermatid cells of infertile men [39]. Therefore, our results may lend further support to AhR overexpression as a characteristic of infertility in males.

Subjects from urban area are more exposed to both MEHP and PFOA, whereas highest PFOA levels, in both blood and seminal plasma, were quantified in men from rural area. The expression levels of all NRs but PPARγ, were lower in the urban and much lower in the rural areas, compared to the metropolitan area, ranging from about 10- to 500-fold depending on each NR. No differences in expression levels between infertile and fertile men from these areas were measured.

MEHP levels measured in this study are comparable to those in Danish men (7.88 ng/mL) [40].

Our findings indicate a potential inverse correlation between MEHP exposure in all subjects considered and the risk of infertility, although this association disappeared when stratifying by area. DEHP and its active metabolite, MEHP, do impair male reproductive development in laboratory rodents [41] and possibly also human semen quality [42,43,44]. We cannot currently make any hypothesis about whether the observed inverse correlation between MEHP exposure and infertility derives from undetected confounder(s) or bears a relationship with MEHP biological activity and metabolism.

With respect to PFOA, its presence in more than 50% of men from urban and rural areas, but not in metropolitan area, suggests the possible presence of specific environmental source of exposure. Indeed, PFOA is known as contaminant of groundwater and water surface from different sources, hinting to a possible relationship with the water sources used in local activities [45].

Results on PFOA and PFOS levels measured in the semen were quite consistent with those obtained in blood samples: detectable PFOA levels were higher in the rural area compared to the other two areas, in both semen and blood matrices. We found no association between PFOA and PFOS for both blood and semen levels and infertility. Literature data on the correlation of perfluorinated compounds with male infertility or sperm quality are not consistent. Altered sperm parameters in highly exposed men were observed in some studies [14,15], including one performed in Siena, another Italian urban area, where PFOA and PFOS internal levels were comparable to those found in our study. On the other hand, another study found no association between PFOA or PFOS exposure and seminal volume, sperm concentration and motility [46].

Interestingly, in our study a significant correlation between PFOA levels in the semen and in blood was observed in infertile but not in fertile subjects which may be considered belonging to the general population. A previous study conducted on the male general population found no correlation between PFOA’s semen and plasma concentrations [47], thus supporting our observation and suggesting that in infertile men an increased circulating blood level of PFOA may be reflected by an increased concentration in the semen.

One goal of the study was to evaluate NR expression as a potentially suitable and toxicologically relevant biomarker in association with environmental exposures and male infertility.

Indeed, we observed that ERα, ERβ, AR, AhR and PXR expression is correlated positively with BPA levels and negatively with PFOA levels, in both infertile and fertile subjects. In effect, higher BPA levels correspond to higher NRs expression, as observed in the metropolitan area, whereas higher PFOA levels correspond to lower NRs expression, as observed in the rural area. The inverse correlation between PFOA and NRs expression in blood and the observed positive correlation between blood and seminal plasma PFOA levels prompt investigating if the same decrease of NRs expression would occur also in spermatozoa, supporting the role of PFOA in male infertility. Indeed, a decrease or a depletion of at least ERs and AR in spermatozoa have been observed in some male reproductive disorders [41,42].

To our knowledge, only a recent study performed on Italian adult men from two rural communities of Tuscany (Central Italy) investigated BPA levels and expression of ERα, ERβ and AR, plus ESRRα and ESSRRβ (estrogen related receptors alpha, and -beta), in PBMCs; BPA exposure was significantly correlated with ERβ and ESRRα, but not with the other NRs considered [48]. Although the design of that study was different from PREVIENI, since it included only healthy subjects from rural communities, BPA was measured in urine and a different NRs panel was used, the overall evidence points to a link between BPA exposure and increased NRs expression in PBMCs.

Within the PREVIENI project, infertile and fertile subjects were enrolled from the selected three areas: as data on women have been already published [29], we will briefly summarize the main overlaps and differences between men and women per area, intending to perform deeper comparison between them in a further study.

The exposure patterns to BPA and PFOA in men are coherent with those observed in women [29]. Indeed, significant higher BPA levels were detected in subjects living in the metropolitan area, while PFOA was present at significantly higher concentration in subjects from rural and urban areas. The overlap of the results obtained in women and men supports the hypothesis that the general population exposure to BPA and PFOA is strongly influenced by the living environment.

Moreover, statistically higher concentrations of BPA were detected in infertile men and women, compared to fertile subjects, but the association between BPA exposure and infertility was highlighted only for women.

A discrepancy was observed as regards MEHP exposure, since higher levels were observed in men from the urban area and in women from the metropolitan area [29]. This finding might hint to some, yet unidentified, gender-related factor differentially present in living environments.

With respect to biomarkers of effect, both men and women from the metropolitan area showed significant higher ERα, ERβ, AR, AhR and PXR genes expression in PMBCs compared to subjects from the other two areas. However, men and women differed as regards the expression of the NRs in fertile and infertile subjects. In fact, infertile women had higher levels of all the NRs compared to fertile women, whereas infertile men had only higher AhR levels than fertile subjects.

A significant positive correlation between BPA serum levels and the expression of all NRs but PPARγ, was observed in infertile subjects, consistently with previous observations in women [35]. On the other hand, while a significant negative correlation between PFOA and all NRs included in the panel, with the exception of PPARγ, was found in infertile men, in infertile women such negative correlation was observed only with AhR.

Overall, our present data support the relevance of the living environment on the exposure of the population, prompting to investigate the contribution of each main route of exposure related to each ED as well as to a combination of co-occurring EDs.

The highlighted correlations between EDs concentration and the expression of NRs provide a panel of biomarkers of effect related to biomarkers of exposure, thus supporting their use in the frame of biomonitoring studies.

While acknowledging the unavoidable limitations of cross-sectional studies, and uncertainties related to the lack of EDs measurements in environmental matrices from the investigated areas, nevertheless, this study highlights significant results on male exposure to several EDs, the relationship with living environments and its potential impact on fertility.

## 5. Conclusions

Our results reinforce the concept that the living environment mainly determines the pattern of EDs exposure in the population. Furthermore, our study confirms the feasibility of NRs panel as biomarkers of effect in association with biomarkers of exposure linked to particular EDs.

The results support the need to assess the possible link between specific EDs, in particular BPA and PFOA, and male infertility.

## Abbreviation

PFOA: perfluorooctanoic acid
PFOS: perfluorooctane sulfonate
DEHP: di-2-ethylhexyl-phthalate
MEHP: mono-(2-ethylhexyl)-phthalate
BPA: bisphenol A
ERα: estrogen receptor α
ERβ: estrogen receptor β
AR: androgen receptor
PPARγ: peroxisome proliferator-activated receptor γ
AhR: aryl hydrocarbon receptor
PXR: pregnane X receptor
